# Identification of candidate flavonoid pathway genes using transcriptome correlation network analysis in ripe strawberry (*Fragaria* × *ananassa*) fruits

**DOI:** 10.1093/jxb/erv205

**Published:** 2015-05-15

**Authors:** Jeremy Pillet, Hao-Wei Yu, Alan H. Chambers, Vance M. Whitaker, Kevin M. Folta

**Affiliations:** ^1^Horticultural Sciences Department, University of Florida, Gainesville, FL 32611, USA; ^2^Gulf Coast Research and Education Center, University of Florida, Wimauma, FL 33598, USA; ^3^Plant Molecular and Cellular Biology Program, University of Florida, Gainesville, FL 32611, USA

**Keywords:** Anthocyanin, coexpresssion, flavonoid, fruit ripening, strawberry, transcriptome.

## Abstract

New modulators of the strawberry flavonoid pathway were identified through correlation network analysis. The transcriptomes of red, ripe fruit from two parental lines and 14 of their progeny were compared, and uncharacterized transcripts matching the expression patterns of known flavonoid-pathway genes were identified. Fifteen transcripts corresponded with putative transcription factors, and several of these were examined experimentally using transient expression in developing fruits. The results suggest that two of the newly-identified regulators likely contribute to discrete nodes of the flavonoid pathway. One increases only *LEUCOANTHOCYANIDIN REDUCTASE* (*LAR*) and *FLAVONOL 3’-HYDROXYLASE (F3’H*) transcript accumulation upon overexpression. Another affects *LAR* and *FLAVONOL SYNTHASE (FLS*) after overexpression. The third putative transcription factor appears to be a universal regulator of flavonoid-pathway genes, as many pathway transcripts decrease in abundance when this gene is silenced. This report demonstrates that such systems-level approaches may be especially powerful when connected to an effective transient expression system, helping to provide rapid and strong evidence of gene function in key fruit-ripening processes.

## Highlight

This work in strawberry fruit predicts new genes relevant to a key metabolic pathway using correlation analysis, and then demonstrates their function in a rapid and effective transient assay.

## Introduction

Commercial strawberry (*Fragaria* × *ananassa*) is a high-value crop, recognized for its sweet flavours, nutrient content and attractive appearance. Consumer analysis has shown that rich red colour and a promise of flavour and nutrition can affect desire to purchase the product (Colquhoun *et al.*, 2012; Mezzetti, 2013). These qualities all share at least a partial basis in the flavonoid pathway, which gives rise to pigments, flavours and healthful compounds. For these reasons, the genetics and biochemistry of the flavonoid pathway have been well studied in strawberry, but our fundamental understanding of pathway regulation is still incomplete.

Strawberries are a rich source of polyphenols, namely flavonoids. These compounds mostly exist as proanthocyanidins, as well as flavonols, anthocyanins and various other phenolics (Carbone *et al.*, 2006; Almeida *et al.*, 2007; Schaart *et al.*, 2013). They are derived through the flavonoid biosynthetic pathway that utilizes a set of enzymatic reactions common to plants, with a few interesting exceptions. For instance, the strawberry *Fra 1* allergen, a PR10-related protein, controls pigment production in ripe strawberry fruits (Muñoz *et al.*, 2010). The challenge is to now identify additional regulators that may control only specific facets of the pathway, which is onerous because the pathway is tightly regulated and a gene missing due to mutation may cause ranging effects and difficult-to-interpret outcomes.

The strawberry fruit is emerging as a model for non-climacteric fruit ripening, and many studies have analysed changes in gene expression during the fruit development and maturation processes (Bombarely *et al.*, 2010; Kang *et al.*, 2013). Linking genes to biological roles in ripening fruits has been facilitated by a robust transient expression system that allows demonstration of gene function (Hoffmann *et al.*, 2006). These assays have been used to identify roles for new genes associated with ripening, including those influencing softening, aromas and pigment accumulation.

Our laboratory has used novel transcriptome profiling methods coupled to analytical chemistry to identify transcripts associated with flavour compounds (Chambers *et al.*, 2014). It is possible that transcriptome data may be used to identify candidate genes relevant to a specific pathway or process using transcriptome co-expression network analysis (TCNA; reviewed in Saito *et al.*, 2008). The method identifies discrete trends in transcript accumulation associated with a given process or pathway (such as the flavonoid pathway), and then identifies other transcripts that follow the same trends. These other transcripts are considered candidates because they are co-expressed with genes contributing to a known process. This ‘guilt by association’ method of gene discovery has identified new regulators in plants, including those related to flavonoid synthesis in *Arabidopsis* (Yonekura-Sakakibara *et al.*, 2007; Yonekura-Sakakibara *et al.*, 2008), tomato (Ozaki *et al.*, 2010), and ripening strawberry (Schaart *et al.*, 2013).

This report uses a TCNA approach to identify new candidates that affect specific nodes of the flavonoid pathway, using a segregating population of octoploid strawberry (*Fragaria* × *ananassa*) plants. The data revealed co-expression relationships between common flavonoid pathway genes and allowed the identification of coincident candidates, focusing on transcription factors potentially playing a role in ripening-related processes. A subset of the candidates was either silenced or overexpresssed in transient fruit assays, and revealed specific mechanisms of action in the regulation of discrete pathway functions. This work couples bioinformatics-based network analysis with a robust transient assay to functionally identify new regulators of flavonoid pathway genes.

## Materials and methods

### Transcriptome analysis

Transcriptome analyses were performed based on the genetic cross described in Chambers *et al.* (2014). Briefly, *Fragaria* × *ananassa* cultivars ‘Mara des Bois’ and ‘Elyana’ were crossed, and fruits from 14 F1 field-grown plants were harvested at a uniform red-ripe stage. RNA extraction from whole red fruits and RNA-seq protocols are described in Chambers *et al.* (2014).

### Plant material, transient assays

Two different cultivars were used for transient fruit assays. Overexpression was performed in *Fragaria* × *ananassa* ‘Strawberry Festival’ (‘Festival’), while silencing was performed in ‘Elyana’. These cultivars have been noted for robust overexpression or silencing responses in the agroinfiltration method (J. Pillet, unpublished). For the developmental series, ‘Festival’ fruits were harvested at the green, white, turning and red stages. Achenes were removed from frozen fruits by grinding them off with a frozen steel file. The resulting powder was collected and analysed separately.

### Correlation analysis

Correlation tests were performed using Pearson product-moment correlation coefficient (Pearson’s *r*) with a two-tailed test and the *P*-value was established for each comparison with 11 degrees of freedom. Correlations were considered positive when Pearson’s *r*>0.65 and *P*<0.05.

### Plasmid construction

For *FaTCP11* (*gene09614*) and *FaPCL1-like* (*gene17673*) overexpression constructs, full-length sequences were amplified from strawberry cv. ‘Elyana’ cDNA using specific primers (see Supplementary Table S1) and cloned into the gateway vector pK7WG2D.1. Silencing of *FaSCL8* (*gene13212*) was accomplished by amplifying a 330bp sequence from cv. ‘Elyana’ cDNA and inserting it into pHELLSGATE12 using gateway technology. Primers used are listed in Supplementary Table S1.

### Transient expression assay

Transient expression in strawberry fruit by agroinfiltration was performed according to Hoffmann *et al.* (2006). Briefly, the *Agrobacterium tumefaciens* strain AGL0 (Lazo *et al.*, 1991) containing the overexpression or silencing plasmid, was grown at 28°C in Luria–Bertani (LB) medium with appropriate antibiotics. When the culture reached an OD600 of ~0.8, *Agrobacterium* cells were harvested and resuspended in a modified MacConkey (MMA) medium (Murashige and Skoog salts, 10mM morpholine ethanesulphonic acid pH 5.6, 20g l^-1^ sucrose). The *Agrobacterium* suspension was then injected into multiple sites over the entire fruit while it was still attached to the plant ~14 d after pollination using a sterile 1ml hypodermic syringe. The fruits were harvested 3–7 d after injection, the day they turned fully red. Fruits at the same developmental stage were used as control. Control for the agroinfiltration effect on the flavonoid pathway was tested using a control plasmid containing the GUS gene and a comparable injection protocol (Supplementary Fig. S1).

### qRT-PCR

Quantitative real-time PCR expression analysis was carried out using the StepOnePlus™ Real-Time PCR System (Applied Biosystems®). Reaction mixes (20 μl) were prepared, which included 10 μl of EvaGreen 2X qPCR MasterMix-ROX (ABM), 0.2mM of each primer and 1 μl of diluted cDNA. Gene transcripts were quantified with normalization to *FaCHP1* as a stable reference transcript (Clancy *et al.*, 2013). All biological samples were tested in triplicate, and SD values of the means were calculated using standard statistical methods. The 2-ΔΔCT method was used for quantification of the transcript expression (Schmittgen and Livak, 2008). Specific annealing of the oligonucleotides was controlled by dissociation kinetics performed at the end of each PCR run. The efficiency of each primer pair was measured on a PCR product serial dilution. Quantitative real-time PCR primer sequences are listed in Supplementary Table S1.

### Availability of supporting data

The RNAseq reads have been deposited into the NCBI Short Read Archive and are accessible under project SRP039356 (http://www.ncbi.nlm.nih.gov/sra/?term=SRP039356). Data represent all reads from parental lines (‘Mara des Bois’ and ‘Elyana’) as well as reads from individual F1 plants.

## Results

### RNA-seq analysis of the ‘Elyana’ × ‘Mara des Bois’ F1 population

‘Mara des Bois’, a 1990’s cultivar with a unique aromatic profile, and ‘Elyana’, a Florida cultivar that produces large, flavourful fruits, were crossed and fruits from 14 F1 individuals were harvested at a uniform fully-expanded, red-ripe stage. An average of 11.7 million clean reads were produced per genotype with a maximum of 20.3 and 19 million reads for the parents ‘Mara des Bois’ and ‘Elyana’, respectively (Table 1). The reads were mapped against the *Fragaria vesca* reference genome, a diploid strawberry genome that shares a common ancestor with one of the octoploid strawberry subgenomes (reviewed in Folta and Davis, 2006). Because of the potential differences between the reads in the octoploid and the reference diploid, analyses were performed at lower stringency (see Materials and Methods). An average of 85.5% of each genotype’s reads were mapped to the reference genome, averaging just over 16 000 expressed genes per genotype in the ripe fruit. Only transcripts with an RPKM >0.5 were considered expressed. Interestingly, the number of transcripts expressed is relatively consistent within the population, representing 15 358 to 16 903 genes. However, important variation in transcript expression was identified when the transcriptome from each progeny was compared to the transcriptomes of the parental lines. For example, some genotypes like P103 have 5673 genes that are differentially expressed compared to ‘Mara des Bois’ and the progeny P193 have 6678 genes differentially expressed compared to ‘Elyana’ (differentially expressed is defined as >2-fold RPKM). On average, 1712 and 3232 genes were more expressed in a given progeny’s transcriptome relative to ‘Mara des Bois’ and ‘Elyana’, respectively, while 2107 and 1533 were more abundant in the parents. These variations were used to build a hierarchical clustering of the population (Supplementary Fig. S2). Global analysis of these variations showed the absence of general trends between the samples and confirmed the apparent homogeneity of the dataset. This allowed investigation of possible correlations among genes expressed in specific biosynthetic pathways.

**Table 1.** RNA-seq global analysis of the ‘Mara des Bois’ × ‘Elyana’ F1 population

**Genotype**	**Nb reads (Millions**)	**% mapped on the *F.vesca* genome**	**Nb expressed genes**	**DEG+ / ‘Mara des Bois’**	**DEG- / ‘Mara des Bois’**	**DEG+ / ‘Elyana’**	**DEG- / ‘Elyana’**
**‘Mara des Bois’**	20.3	86.2	16 421	X	X	3190	1329
**‘Elyana’**	19	84.7	15 358	1319	3190	X	X
**P006**	11.6	86.1	15 975	907	1726	2746	1488
**P024**	10	85.1	16 612	2382	2049	4081	1746
**P037**	8.6	86	16 579	1985	1718	3849	1430
**P042**	10.5	85.7	15 637	881	2341	2656	2186
**P051**	11	87.3	15 833	1344	2268	2268	1525
**P089**	12.2	86.1	16 254	1179	1722	3344	1573
**P091**	12.1	84.3	15 620	1177	2521	1326	950
**P093**	14.2	85.9	16 903	2340	1401	4721	1458
**P098**	10.7	85	16 249	1930	1891	3163	1382
**P103**	8.4	84.5	16 292	2869	2804	3543	1611
**P152**	11.7	86.3	16 496	2248	1904	3595	1666
**P193**	6.9	85.5	16 719	2181	1620	4847	1831
**P203**	7.9	83.5	16 121	1783	2404	2059	1010
**P204**	12.5	86.3	16 028	1149	2042	3092	1805
**Average**	**11.7**	**85.5**	**16 194**	**1712**	**2107**	**3232**	**1533**

DEG+, Differentially more Expressed Genes; DEG-, Differentially less Expressed Genes

### Correlation analysis of the flavonoid pathway

**Fig. 1.**
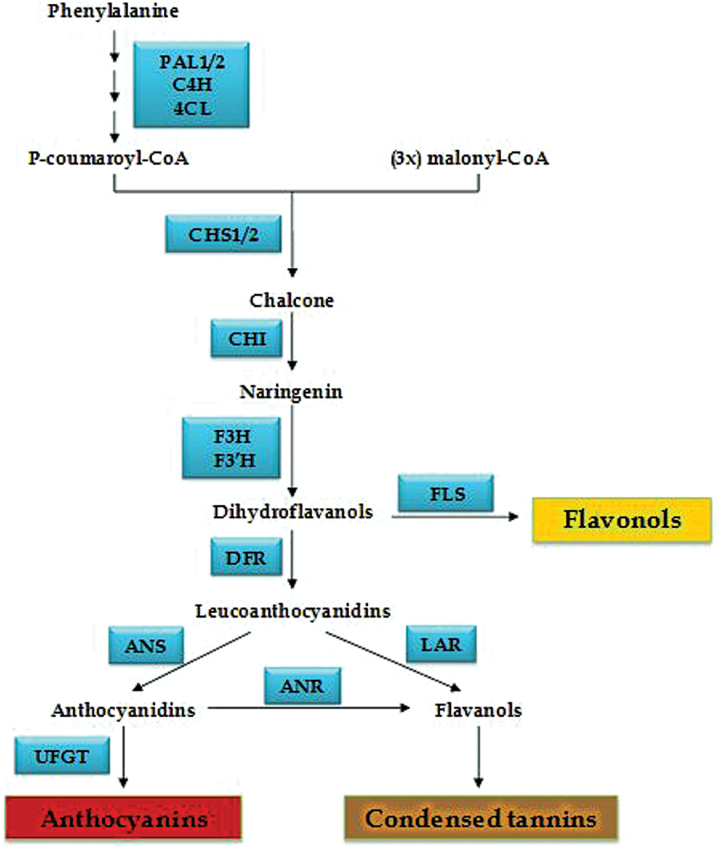
Simplified flavonoid biosynthetic pathway. PAL, Phenylalanine ammonia lyase; C4H, cinnamate-4-hydroxylase; 4CL, 4-coumaroyl:CoA-ligase; CHS, Chalcone synthase; CHI, Chalcone isomerase; F3H, Flavanone 3 hydrolase; F3’H, Flavanone 3’ hydrolase; FLS, Flavonol synthase; DFR, Dihydroflavanol reductase; LAR, Leucoanthocyanidin reductase; ANR, Anthocyanidin reductase; ANS, Anthocyanidin synthase; UFGT, UDP-Glucose flavonoid glycosyl transferase.

The phenylpropanoid pathway, and more specifically the flavonoid pathway, is a well-studied biosynthetic pathway that includes several enzymes that catalyse production of different families of molecules such as the flavonols, the condensed tannins and the anthocyanidins (Fig. 1). The core of this analysis measured transcripts from 15 genes known to be part of these pathways. These include the early and general phenylpropanoid genes such as the *PHENYLALANINE AMMONIA LYASE (PAL*) and some family-specific genes like the *UDP-GLUCOSE FLAVONOID GLUCOSYL TRANSFERASE (UFGT*) that leads to anthocyanin synthesis. Two isoforms of *PAL* and two of *CHALCHONE SYNTHASE (CHS*) were also included.

**Fig. 2.**
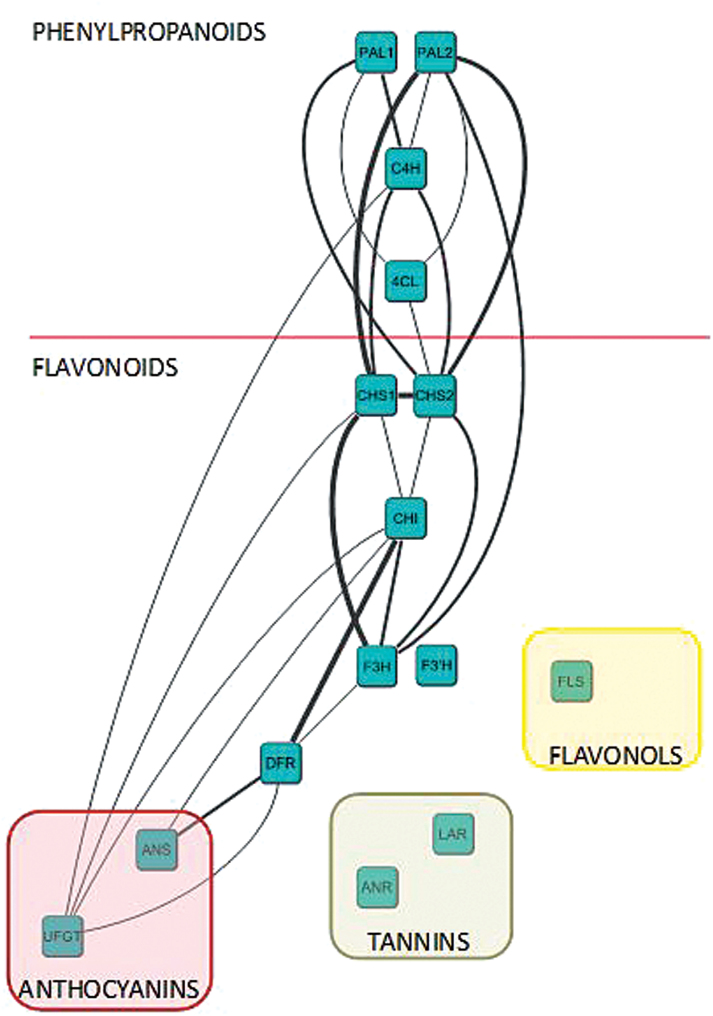
Expression profile correlation analysis of the flavonoid biosynthetic pathway in the ‘Mara des Bois’ × ‘Elyana’ F1 population. Correlations are considered positive when Pearson’s *r*>0.65 (*P*<0.05). The thicker the line the more close to 1 the correlation is. PAL, phenylalanine ammonia lyase; C4H, cinnamate-4-hydroxylase; 4CL, 4-coumaroyl:CoA-ligase; CHS, chalcone synthase; CHI, chalcone isomerase; F3H, flavanone 3 hydrolase; F3’H, flavanone 3’ hydrolase; FLS, flavonol synthase; DFR, dihydroflavanol reductase; LAR, leucoanthocyanidin reductase; ANR, anthocyanidin reductase; ANS, anthocyanidin synthase; UFGT, UDP-glucose flavonoid glycosyl transferase.

Correlations in the expression profiles within this set of genes were calculated, and positive correlations between genes (Pearson’s *r*>0.65, *P*<0.05) were identified (Fig. 2). This analysis reveals that transcript levels for each of the genes in this pathway seem to share strong correlation with at least three or four other genes in the pathway. These findings suggest a strongly connected and co-regulated pathway, consistent with what is already known about the relationships between these genes in other plants, including *Arabidopsis* (Yonekura-Sakakibara *et al.*, 2008) and tomato (Ozaki *et al.*, 2010). Some key genes can also be identified, including two *CHS* isoforms (*CHS1, CHS2*) and *CHI (CHALCONE ISOMERASE).* These transcripts share six, seven and six positive correlations with other genes, respectively. Even when positive correlations were found between either *PAL1* and *PAL2* and the same other genes, no correlation between the two PAL isoforms was detected, suggesting different roles or, more likely, different regulation processes for the two isoforms. Furthermore, the global correlation pattern is in agreement with the metabolic flux occurring in the ripe fruit. Indeed, positive correlations are only found between genes that are involved in the anthocyanin pathway and no correlation were established with genes like *LAR* and *ANR* that are specific for the synthesis of tannins or *FLS* for the flavonols. No correlation was found for the F3’H gene suggesting the implication of this enzyme in processes other than anthocyanin synthesis in ripening strawberry fruit.

### Identification of candidate flavonoid pathway transcription factors from transcriptome coexpression network analysis

Based on the finding that flavonoid pathway genes share expression patterns matching known genes in the pathway, we formulated the hypothesis that any gene that shared at least four positive expression correlations with known flavonoid pathway genes is likely to be functionally connected to the pathway. Using Mapman BINs, 1897 putative transcription factor (TF) homologues were selected, and their individual transcript accumulation patterns were compared to the expression profiles of the flavonoid pathway transcripts. Only 15 TF transcripts showed four or more positive correlations (Pearson’s *r*>0.65, *P*<0.05) with transcripts from the flavonoid pathway (Table 2). Several different families are represented, including MYB, bHLH and zinc finger TFs. Among these TFs, two have already been shown to participate in the regulation of the flavonoid pathway. *FaMYB1* has four positive correlations and plays a role in repression of anthocyanin synthesis (Aharoni *et al.*, 2001). Schaart *et al.* (2013) showed that *FabHLH3*, the strawberry orthologue of *Arabidopsis TT8*, acts as a regulator of proanthocyanindin synthesis when combined with *FaMYB9/FaMYB11*. These results show that the approach is robust, as it identifies known regulators, supporting our hypothesis that other co-regulated transcripts may directly affect this pathway.

**Table 2.** List of the candidate transcription factors that shared at least four positive correlations with the flavonoid pathway

**Nb positive correlations**	**Gene ID**	**RPKM max**	**RPKM min**	**Gene description**	**Publication**
6	*gene04812*	42.34	23.71	Zinc finger (C2H2 type) family protein	
6	*gene17673*	77.03	14.39	Myb family transcription factor	
6	*gene18718*	191.98	78.40	*RPL* (*REPLUMLESS*); DNA binding / sequence-specific DNA binding / transcription factor	
5	*gene09614*	34.13	12.99	TCP family transcription factor, putative	
5	*gene10501*	30.05	7.10	*MYC2;* DNA binding / transcription activator/ transcription factor	
5	*gene16487*	61.39	28.59	Transcription regulator	
**5**	*gene27827*	34.26	12.54	*FabHLH3*	Schaart *et al.*, 2013
4	*gene01828*	379.42	104.35	Trihelix DNA-binding protein, putative	
4	*gene03631*	47.28	5.48	*SGA2*	
**4**	*gene09407*	55.15	26.82	*FaMYB1*	Aharoni *et al.*, 2001
4	*gene13212*	1074.20	378.93	Scarecrow-like transcription factor 8 (*SCL8*)	
4	*gene16801*	24.05	11.79	*ZFN1* (*ZINC FINGER PROTEIN 1*); DNA binding / nuclease/ nucleic acid binding	
4	*gene18815*	26.44	15.70	Myb family transcription factor	
4	*gene27007*	46.30	11.71	Zinc finger (CCCH-type) family protein	
4	*gene31788*	50.70	16.60	Proline-rich family protein	

Correlations are considered positive when Pearson’s r>0.65 (*P*<0.05)

The maximum number of positive correlations identified is six with *gene04812* (a zinc finger TF), *gene17673* (a MYB TF) and *gene18718* (which is the homologue of *Arabidopsis* RPL; Roeder *et al.*, 2003). A bHLH TF identified as a *MYC2*-like factor (gene10501) and another yet uncharacterized MYB TF (*gene18815*) also show strong correlations. Another interesting candidate is *gene13212* due to its relatively high expression (RPKM between 379 and 1074). This transcript is most similar to *Arabidopsis SCARECROW-LIKE 8* (*SCL8*). Three candidate genes were selected for comprehensive expression analysis. The first was *gene17673*, predicted to encode a putative MYB TF. This gene shared positive correlations with PAL1/2, C4H, 4CL and CHS1/2 (Table 3). The second selected candidate is *gene13212*. This transcript accumulates to relatively high levels compared to others and shared correlations with PAL2, CHS1/2 and F3H. The third one is *gene09614* because evidence from other plants indicates interaction of TCP family members with flavonoid pathway regulation.

**Table 3.** Positive correlations observed for the flavonoid-associated genes and their RPKM values

	**FaTCP11**	**FaPCL1-like**	**FaSCL8**	**RPKM min**	**RPKM max**	**Average RPKM**
*PAL1*	-	0.79	-	171	525	343
*PAL2*	0.81	0.76	0.72	84	207	155
*C4H*	-	0.74	-	34	172	91
*4CL*	0.78	0.67	-	272	935	608
*CHS1*	0.78	0.72	0.80	500	1442	1079
*CHS2*	0.78	0.81	0.75	831	2518	1791
*F3H*	0.75	-	0.69	661	2209	1570

### Candidate gene prioritization

**Fig. 3.**
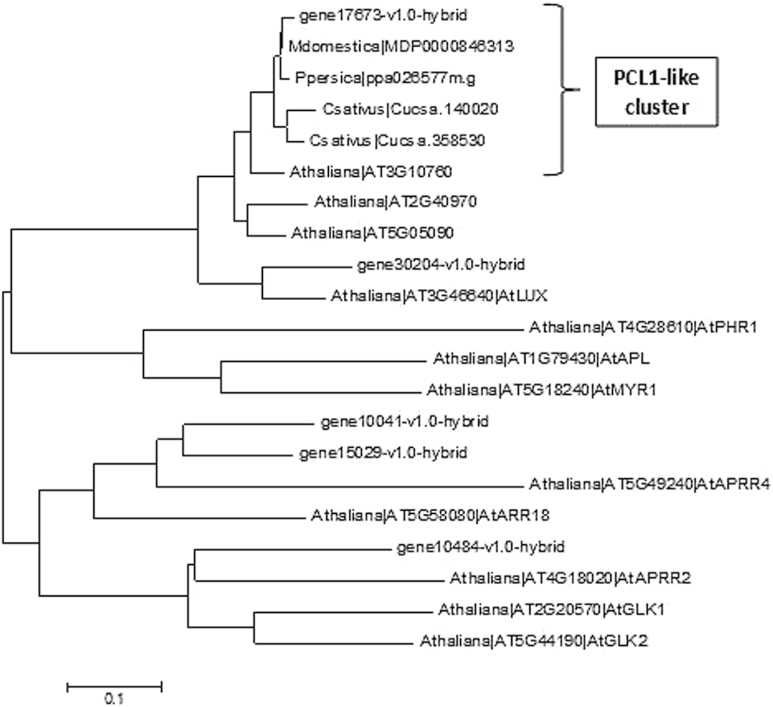
Phylogenetic analysis using the Neighbor–Joining method and based on closest amino acid sequences of gene17673 from different plant species. Bootstrap values are based on 1000 replicates. Bars, 0.1 amino acid substitutions per site.

Three candidates (gene09614, gene17673 and gene13212) were selected based on potential inferred roles in ripening or due to their expression levels. Based on sequence and domain similarity compared to *Arabidopsis*, *gene09614* was named *FaTCP11.* Phylogenetic analysis of *gene17673* reveals that this gene belongs to a cluster of PCL1-like genes (Fig. 3). Closest orthologues from related species such as *Malus domestica, Prunus persica* and *Cucumis sativus* were used for cluster analysis and also family genes from *Arabidopsis thaliana*. Based on this analysis, we named *gene17673* ‘*PCL1-like’*. The transcript noted as *gene13212* is most similar to *AtSCL8* in *Arabidopsis*, a member of the SCARECROW-LIKE gene family, and was named ‘*FaSCL8*’.

### Analysis of expression patterns through ripening process

**Fig. 4.**
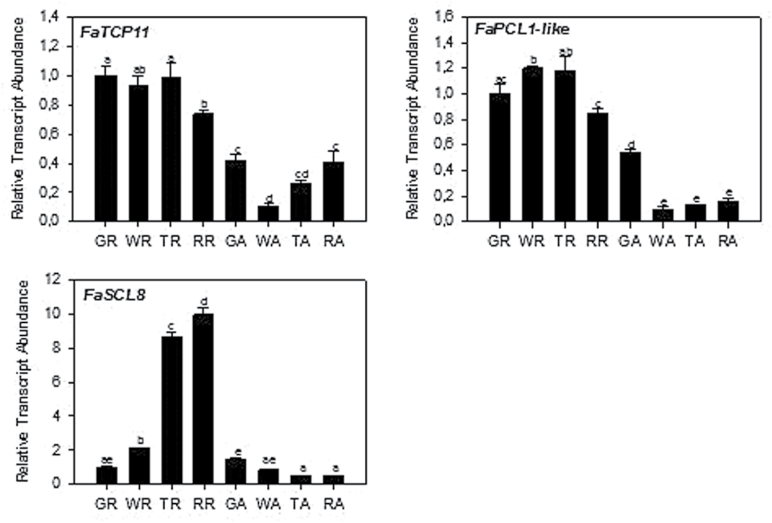
Expression patterns of *FaTCP11*, *FaPCL1-like* and *FaSCL8* during fruit development. GR, green stage receptacle; WR, white stage receptacle; TR, turning stage receptacle; RR, red stage receptacle; GA, green stage achenes; WA, white stage achenes; TA, turning stage achenes; RA, red stage achenes. Significance was measured by a one-way ANOVA with *P*<0.001.

To further infer potential function, candidate transcript accumulation was measured in the receptacle and achenes in response to ripening. In the receptacle, *FaTCP11* and *FaPCL1*-like expression is stable during development and decreases during the red stage (Fig. 4). In the achenes, both transcripts are present at their highest levels at the green stage, and then the expression level decreases with a slight increase later in maturity. On the contrary, *FaSCL8* expression shows an induction in the receptacle coincident with ripening. *FaSCL8* is less expressed in achenes compared to the other flavonoid-associated genes.

### Functional analysis during transient expression

**Fig. 5.**
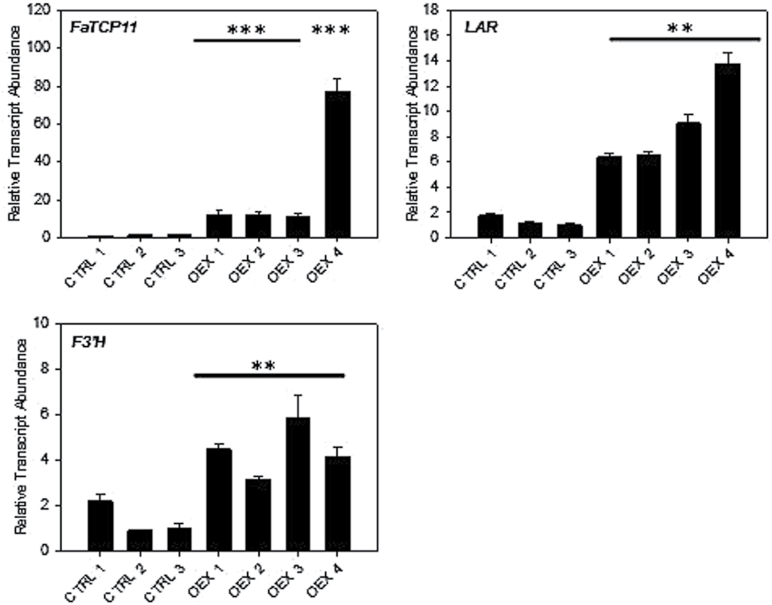
Expression profiles in agro-infiltrated fruits overexpressing *FaTCP11*. Significance was measured by taking into account the relative transcript accumulation in all of the independent fruits compared to all controls. **, *P*<0.01; ***, *P*<0.001. F3’H, flavanone 3’ hydrolase; LAR, leucoanthocyanidin reductase.

The effects of misexpression of the candidate transcripts were assessed using transient expression assays in fruit. Agro-infiltration of maturing strawberry fruit has been a useful tool in defining gene contributions to ripening (Hoffmann *et al.*, 2006). First, fruits were inoculated with constructs unrelated to the flavonoid pathway to ensure that the infiltration process alone would not affect the flavonoid pathway (Supplementary Fig. S2). Next, *FaTCP11* and *FaPCL1-like* were overexpressed in ‘Strawberry Festival’. Overexpression assays were performed because these transcripts were expressed at low levels in developing fruit. *FaTCP11* was overexpressed 10-fold higher in three independent fruits (OEX 1, 2 and 3) and more than 70-fold higher in one fruit (OEX 4) compared to control fruits (Fig. 5). Analysis of the flavonoid gene expression profiles clearly showed that this gene has an effect on the expression of *LAR* which is induced 6–9-fold in OEX 1, 2 and 3 and >13-fold in OEX 4. *FaTCP11* has also an effect on *F3’H* as its expression is slightly induced in the agro-infiltrated fruits. These results suggest a putative, yet specific, role of *FaTCP11* in the synthesis of flavan-3-ols.

**Fig. 6.**
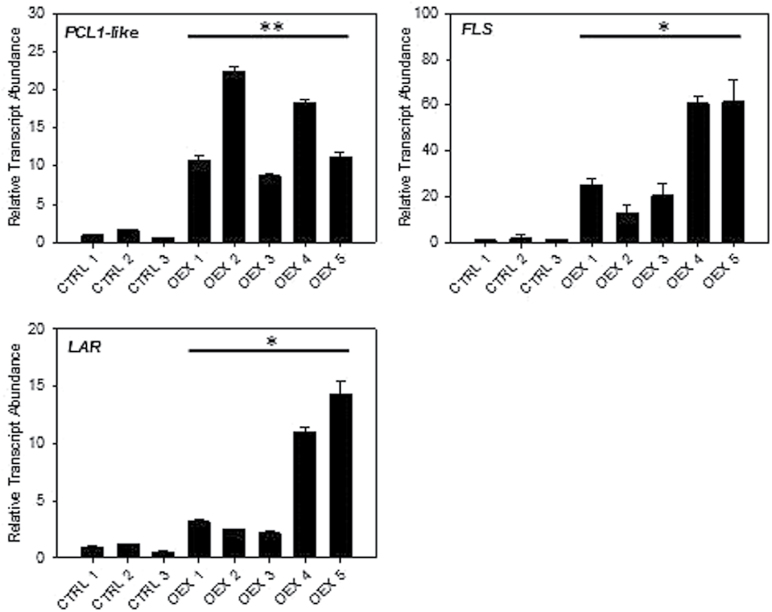
Expression profiles in agro-infiltrated fruits overexpressing *PCL1-like*. Significance was measured comparing expression levels in construct-containing, independent fruits compared to all controls. *, *P*<0.05; **, *P*<0.01. FLS, flavonol synthase; LAR, leucoanthocyanidin reductase.

Transient overexpression of *FaPCL1-like* resulted in transcript levels 10–20-fold higher compared to control fruits (Fig. 6). This overexpression caused a 10–60-fold induction of *FLS*, and suggests a role of this TF in flavonol synthesis. However, overexpression of *FaPCL1-like* also induces the expression of *LAR* from 2–14-fold, and connects this TF to tannin synthesis.

**Fig. 7.**
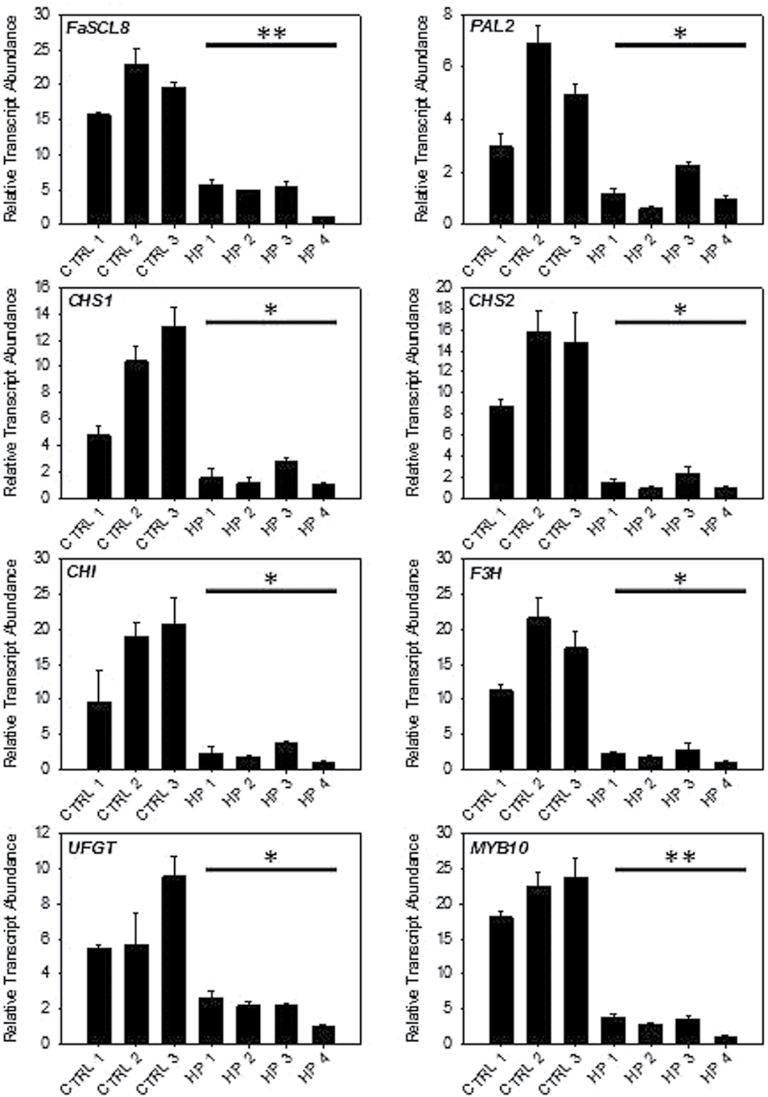
Decreased transcript accumulation in agro-infiltrated fruits with *FaSCL8* silencing. Significance was measured by taking in account all the independent fruits compared to all controls. * *P*<0.05; **, *P*<0.01. PAL, phenylalanine ammonia lyase; CHS, chalcone synthase; CHI, chalcone isomerase; F3H, flavanone 3 hydrolase; UFGT, UDP-glucose flavonoid glycosyl transferase.

**Fig. 8.**
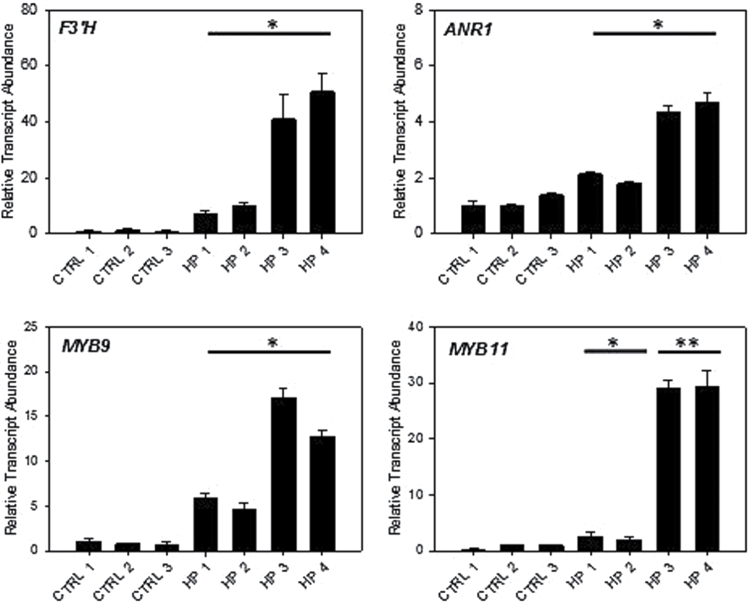
Induced expression profiles in agro-infiltrated fruits with *FaSCL8* suppression. Significance was assessed from the means and variation from the fruits treated with an experimental construct compared to controls. *, *P*<0.05; **, *P*<0.01. F3’H, flavanone 3’ hydrolase; ANR, anthocyanidin reductase.

The last candidate gene analysed was *FaSCL8*. Due to its high expression levels in ripe fruit, RNA silencing was used for functional analysis. Transcript levels were suppressed 3-fold (compared to WT) in three independent fruits (HP 1, 2 and 3) and >15-fold lower in HP 4 (Fig. 7). Silencing *FaSCL8* significantly results in lower transcript accumulation of *PAL2*, *CHS1*, *CHS2*, *CHI*, *F3H* and *UFGT*. It also inhibits the expression of *MYB10*, a TF that regulates the expression of most of the genes involved in anthocyanin production in ripened fruit receptacles. Surprisingly the silencing of *FaSCL8* also results in increased transcript accumulation from *F3’H* and *ANR1,* suggesting another role of this TF in the regulation of the pathway (Fig. 8). Furthermore it appears that *FaSCL8* can influence the expression of *MYB9* and *MYB11*, TFs that can regulate the tannin production in strawberry fruit.

## Discussion

While individual methods in plant functional genomics provide new opportunities for analysis, they are especially effective in combination. In this report we couple the predictive power of transcript co-expression network analysis to the rapid and informative methods of transient expression in ripening strawberry fruits. The results show that new candidates associated with a given pathway or process may be both rapidly identified and functionally tested for evidence of participation in the process being studied. While analyses in transgenic plants usually provide the most accurate and comprehensive description of gene function, the development of transgenic strawberry plants, while relatively fast, still requires almost a year from construct to fruit to analyse. The use of the transient fruit system produces primary evidence of gene function, and allows prioritization of candidates to integrate into transgenic plants for further analysis.

Ripening strawberries were examined from two contrasting parental lines and 14 of their progeny. Using this method, variation in transcript abundance between progeny and parents enabled identification of regulatory gene candidates from network analysis. In other words, if one genotype produced red ripe berries with the flavonoid pathway induced, other transcripts associated with the pathway also tended to accumulate. If transcripts were not found in a given genotype, it tended to be in concert with similar expression of other flavonoid pathway genes. These trends should be expected because of the highly coordinated expression in the pathway. This analysis tests the hypothesis that transcripts common to a given a network tend to be expressed together, so novel transcripts correlating with those of a given pathway may actually perform a function in that pathway of interest.

*Fragaria* × *ananassa* possesses an octoploid genome, so any transcriptome assessment will likely contain transcript sequences arising from homoeologous genes. The sequencing of the transcriptome derived from the four sub-genomes could provide useful information concerning regulation between sub-genomes or allelic hierarchy for a given gene. One potential drawback of this analysis is that the diploid reference genome was used to build transcript models, which potentially leads to the loss of valuable information. However by allowing some flexibility in matching reads to the genome we were able to map the vast majority of the reads, meaning that the transcripts from different homoeologous alleles were merged into a common transcript (Table 1). This approach allowed more data to be merged into better models, and improved statistical support for measuring expression correlations.

The findings herein identify new regulators for facets of the flavonoid pathway, identified through correlation network analysis (Fig. 2). These results suggest that the pathway relies on a highly regulated mechanism that requires coordinated participation of many genes that control the flux through the pathway, shunting to branch points and controlling the overall amplitude of pathway products. Such overarching pathway control is described as a metabolon. A metabolon is a temporary structural-functional complex formed between sequential enzymes of a metabolic pathway, connected by non-covalent interactions and structural elements of the cell such as integral membrane proteins or proteins of the cytoskeleton. Two models are actually proposed for the flavonoid pathway. The first one suggests a linear construction, with one enzyme linked to a previous and a following one (Jørgensen *et al.*, 2005; Lepiniec *et al.*, 2006). The second model refers to a more complex multi-enzymatic process, including the nexus of several enzymes and structural features in a metabolon. In the example demonstrated here, the complex coordination leads to production of anthocyanins and tannins (Ralston *et al.*, 2005). Such complex interactions require concerted regulatory mechanisms in order to facilitate proper production of products resulting from the pathway endpoints.

**Fig. 9.**
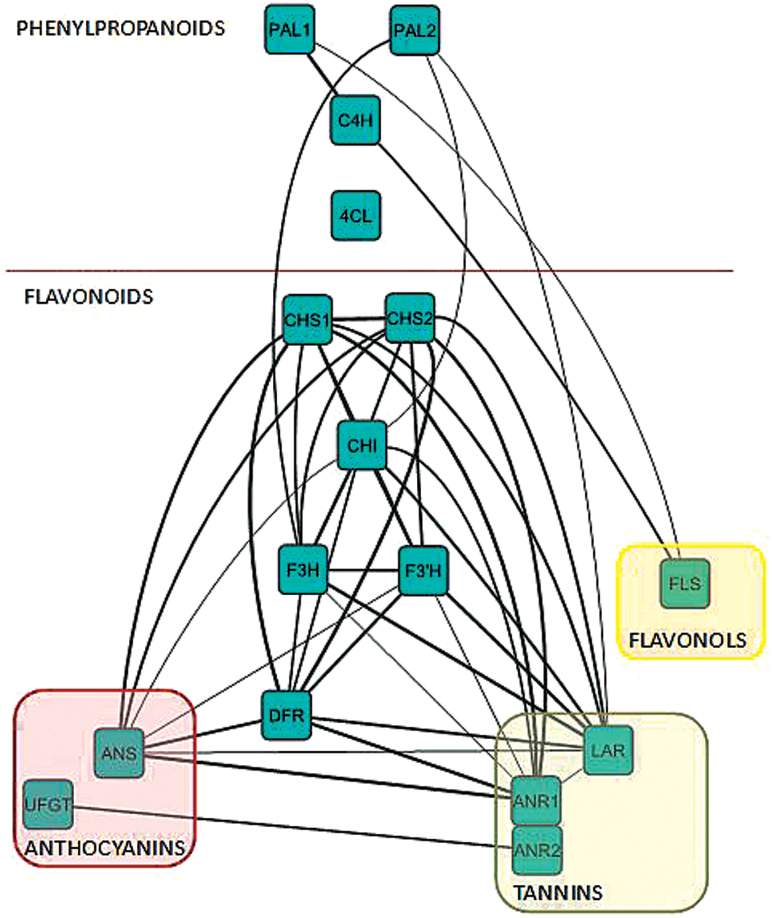
Expression correlation analysis during fruit early development in *Fragaria vesca.* Correlations are considered positive when Pearson’s *r*>0.65 (*P*<0.05). The thicker the line the more close to 1 the correlation is. PAL, phenylalanine ammonia lyase; C4H, cinnamate-4-hydroxylase; 4CL, 4-coumaroyl:CoA-ligase; CHS, chalcone synthase; CHI, chalcone isomerase; F3H, flavanone 3 hydrolase; F3’H, flavanone 3’ hydrolase; FLS, flavonol synthase; DFR, dihydroflavanol reductase; LAR, leucoanthocyanidin reductase; ANR, anthocyanidin reductase; ANS, anthocyanidin synthase; UFGT, UDP-glucose flavonoid glycosyl transferase.

The correlations between expressed genes in the flavonoid pathway provide an indirect image of the metabolic flux in the pathway. In our dataset, composed only of ripe fruits, genes that lead to anthocyanin synthesis were the only ones sharing positive correlations. We applied the same method to a public dataset based on the early stages of fruit development in *Fragaria vesca* and we found that the positive correlation patterns lead to tannin synthesis, which matches known metabolic flux observed during early expansion stages (Fig. 9; based on data from Kang *et al.*, 2013). These analyses predict that data from a ripening series may contain evidence of tannin-directed gene expression early that realigns toward anthocyanins the fruit matures.

Because transcript levels change in concert for the pathway, a ‘guide-gene’ approach for the co-expression network analysis has been performed in order to identify ‘guilt-by-association’ genes that could be putatively involved in the flavonoid pathway. This kind of approach has already been successfully used in *Arabidopsis* in order to identify novel regulators of the flavonoid pathway in that species (Yonekura-Sakakibara *et al.*, 2008). The present study focused on transcription factors (TFs), and 15 candidates were identified that fit the correlation pattern. Two of the 15 TFs identified have already been experimentally shown to be involved in the regulation of this pathway, confirming the approach. Based on annotation and expression profiles, three candidates were selected for further analysis, *FaTCP11*, *PCL1-like* and *FaSCL8*. These transcripts were functionally examined using the transient expression assay in ripening fruits.

Agro-infiltrated fruits overexpressing *FaTCP11* show specific induction of *LAR* and *F3’H* (Fig. 5). This pattern suggests a role of this TF in flavan-3-ols synthesis. *FaTCP11* belongs to TCP family. This family is named after the three first characterized family members *TEOSINTE BRANCHED* (*TB*) 1 from maize, *CYCLOIDEA* (*CYC*) from *Antirrhinum majus*, and *PROLIFERATING CELL FACTORS* (*PCF*s) from rice (Kosugi *et al.*, 1997). The first implication of these genes were established in the regulation of growth and development but then it appears that they were involved in many other process like senescence, circadian rhythms or hormone signaling (reviewed in Martín-Trillo and Cubas, 2010). A recent study in tomato reports the identification and characterization of the entire family present in the tomato genome (Parapunova *et al.*, 2014). Interestingly, some of TCP members in this species seem to participate in ripening-related processes. Furthermore, in *Arabidopsis*, it has been shown that TCP3 can enhance flavonoid biosynthesis by interacting with R2R3-MYB proteins (Li and Zachgo, 2013). Based on those results, we suggest that *Fa*TCP11 plays a role during ripening and in the regulation of the flavan-3-ols synthesis.

*FaPCL1-LIKE*, encoding a MYB TF, was shown to able to specifically modify the expression of *FLS* (Fig. 6). This result suggests a role of this TF in the regulation of flavonol synthesis. The closest *Arabidopsis* homologue is uncharacterized and no functional data have been derived for the *PCL1-like* genes that share the greatest sequence identity. This gene was not identified in co-expression analyses in tomato or *Arabidopsis* flavonoid pathways, so the identification here underscores how this approach may have utility of redefining pathway components specific to different fruits.

Transient expression assays reveals that the TF *Fa*SCL8 has an impact on many transcripts within the flavonoid pathway (Figs 7, 8). The gene is expressed at a high level, so silencing was used to demonstrate function. Most flavonoid pathway genes were significantly repressed. This TF could be considered as a general enhancer of the pathway. However, this enhancement could be indirectly due to the repression of *MYB10* by *FaSCL8* silencing. MYB10 has been shown to regulate the expression of the Early-regulated Biosynthesis Genes (EBGs) and the Late-regulated Biosynthesis Genes (LBGs) genes in anthocyanin production, including those functioning in ripened fruit receptacles (Medina-Puche *et al.*, 2014). This MYB TF is considered as one of the major factors in the regulation of the flavonoid/phenylpropanoid pathway during the ripening of strawberry. Being part of the *SCARECROW* gene family, *Fa*SCL8 is considered to have general roles during development, primarily in response to gibberellins (Pysh *et al.*, 1999), and in this study we show that *MYB10* and *MYB9/11* are among its possible targets or interactors.

Suppression of *FaSCL8* leads to an increase of *F3’H* (Fig. 7) transcript accumulation. As shown previously by the correlation analysis, *F3’H* seems to behave differently from the majority of the flavonoid genes. The effect of *FaSCL8* is consistent with this result. The global repression of anthocyanin genes in the agro-infiltrated fruits did not affect anthocyanin content (data not shown) but the induction of *F3’H* could lead to the synthesis of a different sub-family of anthocyanins. In strawberry the two major classes of anthocyanins are cyanidin and pelargonidin derivatives (Lopes-da-Silva *et al.*, 2002). Cyanidin is F3’H-dependent and pelargonidin is F3’H-independent. The induction of *F3’H* in *FaSCL8*-silenced fruit suggests a modification in the cyanidin-pelargonidin balance that will be the subject of further investigation. Again the action of *FaSCL8* on the pathway seems to be related to another transcription factor. Indeed, the induction of *F3’H* by *FaSCL8* silencing could be due to the induction of *FaMYB9/11*. In conclusion, *FaSCL8* seems to be able to modify the flavonoid genes expression in an indirect way by modulating the expression of known flavonoid regulating TFs. Future work will also include further characterization of these transcripts in stable transgenic lines.

Taken together, the results presented here confirm the applicability of using TCNA to identify transcription factors associated with a given biological process. The strength of using a segregating F1 population in order to identify new candidate genes for a metabolic pathway was clearly demonstrated. Roles for candidate genes were functionally assessed, and raise indications of complex connections between different sub-pathways that will be examined more precisely in transgenic lines. A well-studied pathway was investigated in this report, but the real power in this analysis could be proven by elucidating less well-characterized pathways of interest. This approach could provide a technical solution for resolving pathway components in diverse species of interest.

## Supplementary data

Supplementary data are available at *JXB* online.

Supplementary Table S1. A list of the primer sequences used in this study.

Supplementary Fig. S1. The effect of the agroinfiltration process on the induction of flavonoid pathway genes.

Supplementary Fig. S2. Hierarchical clustering of the ‘Mara des Bois’ × ‘Elyana’ F1 population.

Supplementary Data
