# Supplementary material for: Identification of candidate flavonoid pathway genes using transcriptome correlation network analysis in ripe strawberry (Fragaria × ananassa) fruits
Source: J Exp Bot. 2015 May 15;66(15):4455–67. doi: 10.1093/jxb/erv205 (PMC4507756; doi:10.1093/jxb/erv205)
Supplement: Supplementary Data [file supp_66_15_4455__index.html]

Identification of candidate flavonoid pathway genes using transcriptome correlation network analysis in ripe strawberry (Fragaria × ananassa) fruits — Supplementary Data 

# Identification of candidate flavonoid pathway genes using transcriptome correlation network analysis in ripe strawberry (*Fragaria* × *ananassa*) fruits

## Supplementary Data

Data files

- Supplementary Data - Supplementary Data
