# Supplementary material for: Identification of candidate flavonoid pathway genes using transcriptome correlation network analysis in ripe strawberry (Fragaria × ananassa) fruits
Source: J Exp Bot. 2015 May 15;66(15):4455–67. doi: 10.1093/jxb/erv205 (PMC4507756; doi:10.1093/jxb/erv205)
Supplement: Supplementary Data [file supp_erv205_erv205_supplementaryData_SH.pdf]

Table S1. List of primers used in the study

Gene	Experiment	Primers	Sequences (5'-3')
gene13212	Silencing	Forward	GGGGACAAGTTTGTACAAAAAAGCAGGCTACTCAGTTCCTCTACGAGCTCT
gene13212	Silencing	Reverse	GGGGACCACTTTGTACAAGAAAGCTGGGTGCTTGTGACCCACCACGTTG
gene09614	Overexpression	Forward	GGGGACAAGTTTGTACAAAAAAGCAGGCTTTATGGGCTCCGAGATGGCCCTC
gene09614	Overexpression	Reverse	GGGGACCACTTTGTACAAGAAAGCTGGGTCTAGAATCTCACTTCTCTAGT
gene17673	Overexpression	Forward	GGGGACAAGTTTGTACAAAAAAGCAGGCTTGATGAGGGAAGACGACTCCAAT
gene17673	Overexpression	Reverse	GGGGACCACTTTGTACAAGAAAGCTGGGTTCAATTATCCCCAGTAGGAAA
gene13212	qPCR	Forward	ACCGGGTTTCGGTTTCTATG
gene13212	qPCR	Reverse	CCTGTGGTCAATCATCTTCTTCT
gene09614	qPCR	Forward	GAGATCGACCAACACTAAGGAC
gene09614	qPCR	Reverse	CTCCCTTGTCAGTTGGAAGATT
gene17673	qPCR	Forward	CTGAGGAGCTTATGCCCTTATC
gene17673	qPCR	Forward	GGTATTGGTATTTGGATTGGGATTT
PAL1	qPCR	Reverse	GAAGGACTTGCTTTGGTGAATG
PAL1	qPCR	Forward	GATCTCGGAAAGAACAGCTAGAG
PAL2	qPCR	Reverse	GAGCAGCCATGCTTGTTAGA
PAL2	qPCR	Forward	AGGGAGTAATGTTGTGGTTGAG
C4H	qPCR	Reverse	TGCGCAGAGCTTTGAGTATAA
C4H	qPCR	Forward	TCTTCTCCTTCACCTCCTTACA
4CL	qPCR	Reverse	CGTCAGACTCTATTGCCGATTT
4CL	qPCR	Forward	ATGAAGTTCCGGTGCGTTAG
CHS1	qPCR	Reverse	AGACAAGACATGGTGGTAGTTG
CHS1	qPCR	Forward	AAGTGGGTGATTCTGGACTTG
CHS2	qPCR	Forward	GCTCACATTTCACCTCCTCAA
CHS2	qPCR	Reverse	TCCAGTCGGTAATGTTCAAAGG
CHI	qPCR	Forward	TCGGAGTCTACTTGGAGGATAAG
CHI	qPCR	Reverse	CGATCTCCCTGAAGAACTCAAC
F3H	qPCR	Forward	CTTCATCGTTTCCAGCCATTT
F3H	qPCR	Reverse	ACTGCGTTGTCACATCTCTC
F3'H	qPCR	Forward	CCGTAGCGTCTCAGTTCTTG
F3'H	qPCR	Reverse	ACGAGGTCCTGGTAGTTGTA
DFR	qPCR	Forward	CCGGACTTTCGCCTCTATTT
DFR	qPCR	Reverse	GCTTTCGGATGCTCGTACA
ANS	qPCR	Forward	GGAGAAATGTAGGGAGGAGTTG
ANS	qPCR	Forward	TTCTTGACCCGTTCCATGAG
UFGT	qPCR	Reverse	TGGTCACGATGAGAAGGAAAC
UFGT	qPCR	Forward	GACTCCAAGTTTCCGAAGATGA
ANR1	qPCR	Reverse	TAACCGATGAAGGGAGCTTTG
ANR1	qPCR	Forward	GGCTTGATCATGTCGTTCTCT
LAR	qPCR	Reverse	CATGTATTTGGAGAAGCGCAAG
LAR	qPCR	Forward	GTTGTCATAGTAGGGCCAAGAA
FLS	qPCR	Reverse	GCAGGAGGAGAAAGAGGTTTAC
FLS	qPCR	Forward	CCCTTTCTTCCCTTCCAGTTC
Myb9	qPCR	Reverse	CCGGATCATCCAAGGAAACA
Myb9	qPCR	Forward	CCTAGTGGCCTTGGTTCTTATT
Myb10	qPCR	Forward	TGGCATCATGTTCCTCTCAAA
Myb10	qPCR	Reverse	CTCTGCAAACTCTCCTCTCTTG
Myb11	qPCR	Forward	GATGGTCTTTGATAGCGGGTAG
Myb11	qPCR	Reverse	TGGTGGTTTGTGGTGAGTAAT

Supplementary Figure 1

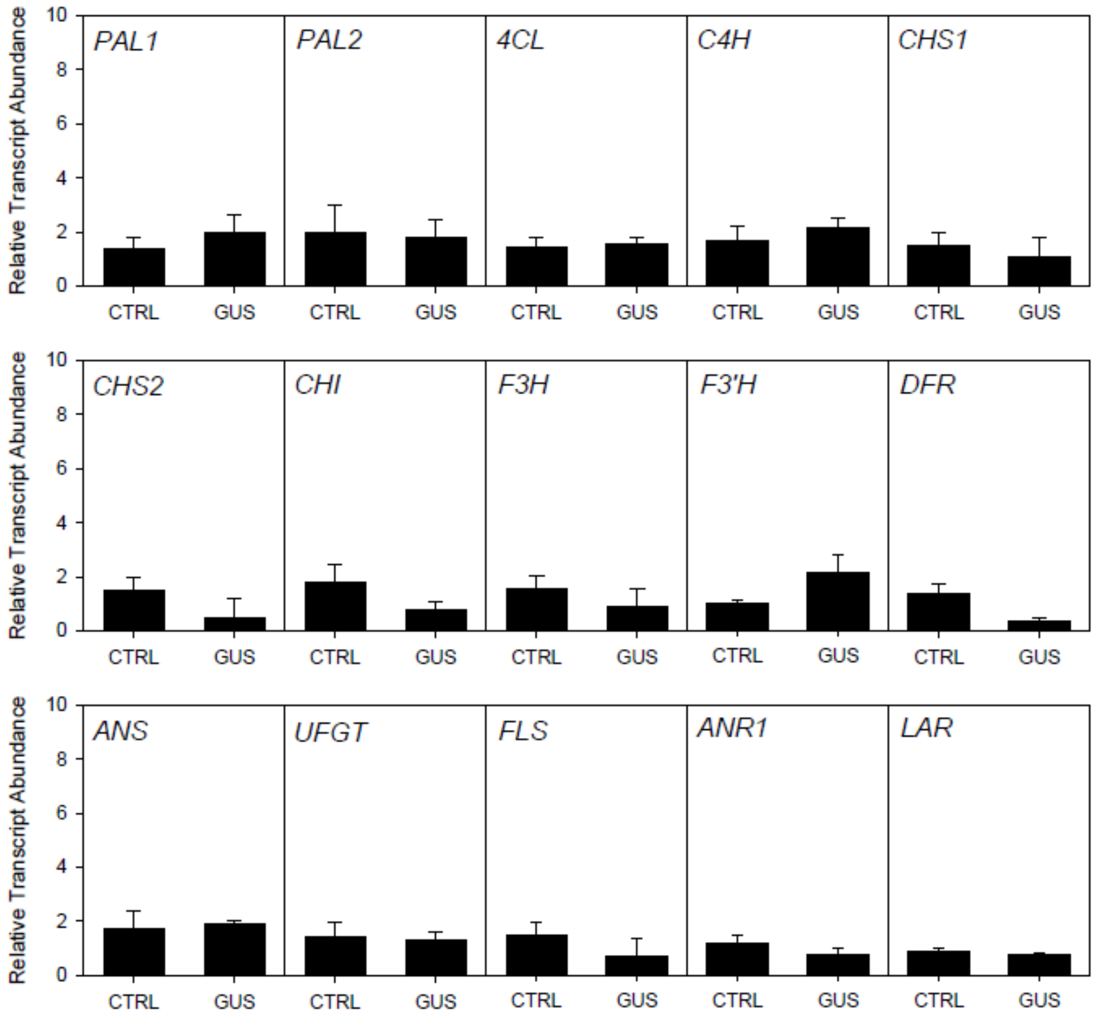

Supplementary Fig. S1. Assessment for effects of the agroinfiltration process on induction of flavonoid pathway genes. Fruits were infiltrated with bacteria bearing a 'GUS' containing sequence. No significant changes were observed.

Supplementary Figure 2

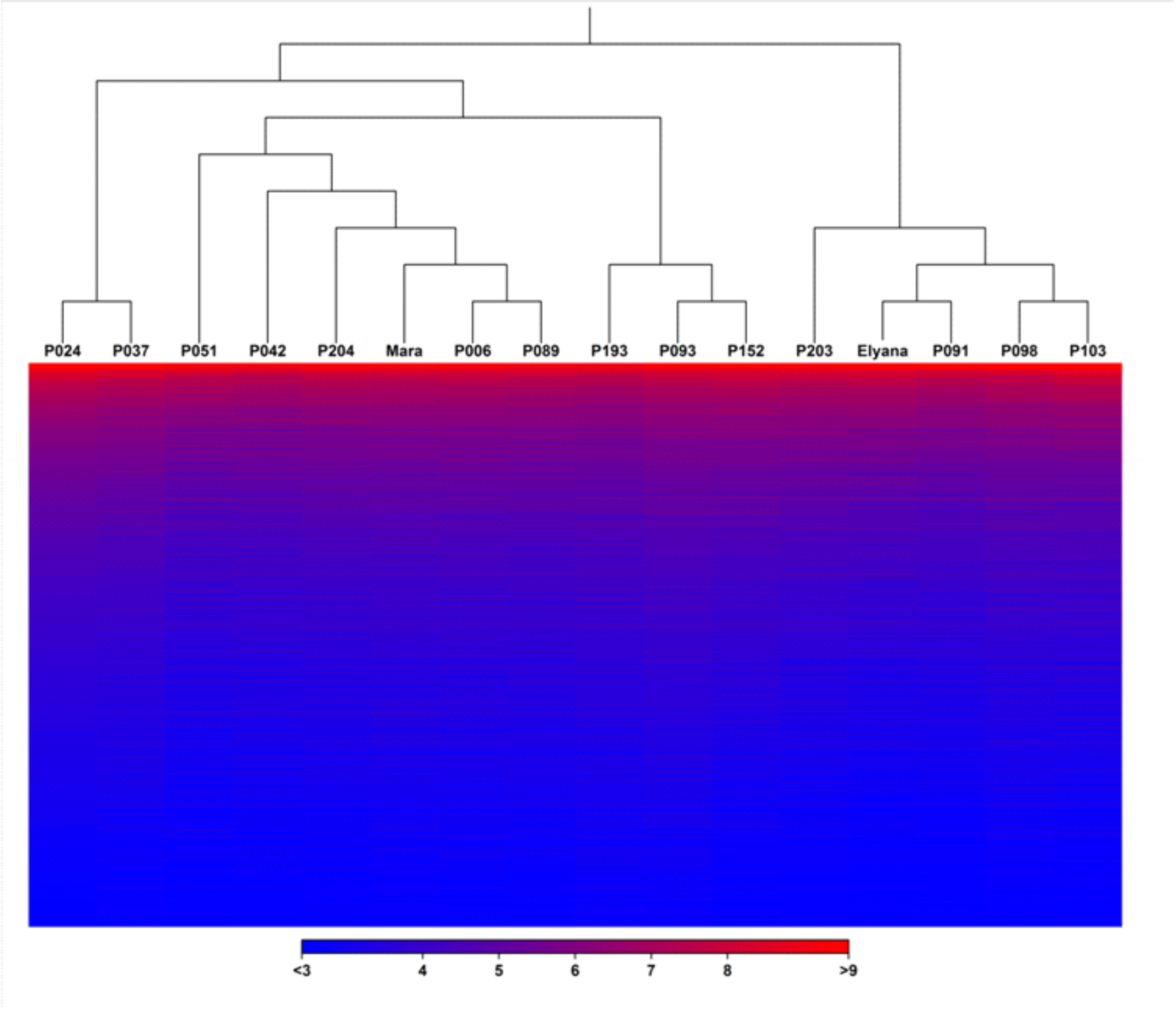

Supplementary Fig. S2. Hierarchical clustering of the 'Mara des Bois' x 'Elyana' F1 population. The dendrogram was assembled based on the transcriptome of each individual, and only expressed genes (RPKM max of 1 in at least one genotype) were included. RPKM values were transformed in Log2. Heirarchical clustering was built using Manhattan distance.
